# Using Natural Language Processing to identify text for qualitative research into the lived experience of people with dementia

**DOI:** 10.1002/alz.093004

**Published:** 2025-01-09

**Authors:** Lily N. Shapiro, William I Bowers, Kelly Ehrlich, Tiina Maripuu, Jenna Mae Peterson, Marlaine Figueroa Gray, Sundary Sankaran, Paul K. Crane, Janelle S Taylor, Robert B Penfold

**Affiliations:** ^1^ Kaiser Permanente Washington Health Research Institute, Seattle, WA USA; ^2^ University of Toronto, Toronto, ON Canada; ^3^ University of Washington, School of Medicine, Seattle, WA USA

## Abstract

**Background:**

Medical records present a rich potential source of information on the lived experiences of people with dementia. These records are extensive and the work of extracting relevant data is labor‐intensive. We sought to determine whether we could use natural language processing (NLP) approaches to sift through medical records to prioritize an enriched subset of notes illuminating the lived experiences of people with dementia.

**Methods:**

We identified an analytic cohort of adults aged ≥ 65 years from the Adult Changes in Thought (ACT) Study, a prospective cohort study of dementia in Seattle, WA. We collected a corpus of 254,532 notes from the electronic medical records (2003‐present) of these participants. The corpus included a range of clinical encounters from five years prior to dementia onset to most recent visit. We implemented an NLP algorithm, pytakes, which extracted concepts from notes in our corpus. The concepts were codes adapted from a prior qualitative study, which we further refined for this study. We used these concepts to curate smaller sub‐corpora for manual, qualitative review.

**Results:**

Starting with 32 unique concepts, pytakes extracted over 1.7 million concepts. 178,042 notes (69.94% of the original corpus) had at least one associated concept. Preliminary review of notes extracted showed acceptable concurrence between the text retrieved and the target concepts. Leveraging the extracted concepts, we curated, then randomly sampled 500 notes for three sub‐corpora, each aimed at a different research question. The first investigates the topics and timings of patient‐ or caregiver‐initiated communications with the healthcare system. The second relates to caregiving needs and networks of people with dementia and how these shift over time. The third revolves around housing situations and transitions of people with dementia. Our qualitative team is examining these sub‐corpora to investigate the lived experience of people with dementia.

**Conclusions:**

NLP tools may be a productive method for identifying materials suited to qualitative inquiry into the lives of people with dementia. This innovative approach can provide access to data that would otherwise be difficult to obtain, offering insight into the lived experiences, care needs, living situations, and social networks of people living with dementia.

